# Barriers to implementing antimicrobial stewardship programs in three low- and middle-income country tertiary care settings: findings from a multi-site qualitative study

**DOI:** 10.1186/s13756-021-00929-4

**Published:** 2021-03-25

**Authors:** Robert Rolfe, Charles Kwobah, Florida Muro, Anushka Ruwanpathirana, Furaha Lyamuya, Champica Bodinayake, Ajith Nagahawatte, Bhagya Piyasiri, Tianchen Sheng, John Bollinger, Chi Zhang, Truls Ostbye, Shamim Ali, Richard Drew, Peter Kussin, Deverick J. Anderson, Christopher W. Woods, Melissa H. Watt, Blandina T. Mmbaga, L. Gayani Tillekeratne

**Affiliations:** 1 Duke University, Durham, NC USA; 2 Duke Global Health Institute, Durham, NC USA; 3 Moi University/Moi Teaching and Referral Hospital, Eldoret, Kenya; 4 Kilimanjaro Christian Medical University College, Moshi, Tanzania; 5 Kilimanjaro Clinical Research Institute, Kilimanjaro Christian Medical Centre, Moshi, Tanzania; 6 Colombo Teaching Hospital North, Ragama, Sri Lanka; 7 University of Ruhuna, Galle, Sri Lanka; 8 Teaching Hospital Karapitiya, Galle, Sri Lanka; 9 Campbell University College of Pharmacy and Health Sciences, Buies Creek, NC USA; 10 Duke Center for Antimicrobial Stewardship and Infection Prevention, Durham, NC USA; 11 University of Utah, Salt Lake City, UT USA

**Keywords:** Antimicrobial Stewardship, Low- and middle-income countries, Qualitative analysis

## Abstract

**Background:**

Antimicrobial resistance has been named as one of the top ten threats to public health in the world. Hospital-based antimicrobial stewardship programs (ASPs) can help reduce antimicrobial resistance. The purpose of this study was to determine perceived barriers to the development and implementation of ASPs in tertiary care centers in three low- and middle-income countries (LMICs).

**Methods:**

Interviews were conducted with 45 physicians at tertiary care hospitals in Sri Lanka (n = 22), Kenya (12), and Tanzania (11). Interviews assessed knowledge of antimicrobial resistance and ASPs, current antimicrobial prescribing practices, access to diagnostics that inform antimicrobial use, receptiveness to ASPs, and perceived barriers to implementing ASPs. Two independent reviewers coded the interviews using principles of applied thematic analysis, and comparisons of themes were made across the three sites.

**Results:**

Barriers to improving antimicrobial prescribing included prohibitively expensive antimicrobials, limited antimicrobial availability, resistance to changing current practices regarding antimicrobial prescribing, and limited diagnostic capabilities. The most frequent of these barriers in all three locations was limited drug availability. Many physicians in all three sites had not heard of ASPs before the interviews. Improved education was a suggested component of ASPs at all three sites. The creation of guidelines was also recommended, without prompting, by interviewees at all three sites. Although most participants felt microbiological results were helpful in tailoring antibiotic courses, some expressed distrust of laboratory culture results. Biomarkers like erythrocyte sedimentation rate and c-reactive protein were not felt to be specific enough to guide antimicrobial therapy. Despite limited or no prior knowledge of ASPs, most interviewees were receptive to implementing protocols that would include documentation and consultation with ASPs regarding antimicrobial prescribing.

**Conclusions:**

Our study highlighted several important barriers to implementing ASPs that were shared between three tertiary care centers in LMICs. Improving drug availability, enhancing availability of and trust in microbiologic data, creating local guidelines, and providing education to physicians regarding antimicrobial prescribing are important steps that could be taken by ASPs in these facilities.

## Background

It is predicted that by 2050, there will be about 10 million deaths due to antimicrobial-resistant organisms every year if strategies are not implemented to curb the rise of resistant organisms globally [1]. Antimicrobial resistance (AMR) is driven in part by the use, and frequently misuse, of antimicrobials in humans and animals [2]. Antimicrobial courses that are either too short or too long in duration, or too broad in spectrum, can lead to the emergence of resistance.

Antimicrobial stewardship is a set of actions at global, national, and individual levels to promote the rational use of antimicrobials in humans, animals, and the environment [3]. Antimicrobial stewardship programs (ASPs) are programs that allow healthcare systems to strategize use of antimicrobials through the implementation of evidence-based interventions. Implementation of ASPs is one of the recommended strategies to decrease the continued rise of AMR. These programs set forth strict guidelines and restrictions for appropriate use [4]. The Centers for Disease Control and Prevention (CDC) published the guidance document “The Core Elements of Hospital Antibiotic Stewardship” to help hospitals develop these programs. The seven core elements of ASPs laid out in this document include hospital leadership commitment, accountability, pharmacy expertise, action, tracking, reporting, and education. While 85% of hospitals in the United States had ASPs that were compliant with the CDC’s seven core elements by 2018, widespread adoption of hospital-based ASPs in low- and middle- income countries (LMICs) has not been accomplished [5].

The World Health Organization (WHO) developed a practical toolkit for ASPs in LMICs [6]. This document provides guidance on how to implement ASPs in LMICs. However, to implement ASPs at a specific site, it is important to comprehend the local context. In some LMICs, antimicrobials are available in pharmacies, or even in markets, without prescriptions. Quality of antimicrobials is often variable in LMICs as well. In a review of generalized challenges facing LMICs in the implementation of ASPs, the main challenges in many resource-limited settings were access to diagnostics, access to quality antimicrobials, knowledge among practitioners, and healthcare facility infrastructure [7].

Our team has been working to support the implementation of ASPs in a context-sensitive manner in Sri Lanka, Kenya, and Tanzania. Our US academic medical center has strong research relationships with tertiary care hospitals in each of these countries. As these are the centers where we are actively working, we felt it would be appropriate to evaluate barriers that are either shared between or unique to these three sites. All three hospitals are at the tertiary care level, allowing for comparison between the countries. In this study, we conducted a qualitative assessment of medical doctors at different levels of training in medical wards in hospitals in each of the three countries to assess receptiveness to ASPs and to elicit opinions on the design of these programs within their health systems. The goal of this study was to identify perceived barriers to the development and implementation of ASPs.

## Methods

This was a qualitative study of physicians to understand their perceptions of current antimicrobial use and receptiveness to ASPs. Individual in-depth interviews were conducted with 45 physicians in 2018 at three tertiary care hospitals in Sri Lanka, Kenya and Tanzania.

### Setting and participants

The hospital in Sri Lanka is a public tertiary care center with 1,800 beds and 10 adult medical wards. This hospital has a microbiology laboratory and radiology services, and provides all medications, testing, and care free of charge to patients. The hospital has a microbiology specialty service consisting of an attending-level microbiologist, microbiology physicians in training, and infection prevention nurses, and can be consulted by other physicians in the hospital for advice regarding antimicrobial prescribing. Pharmacists dispense medications but otherwise are not involved in clinical care and do not provide advice on therapy.

The hospital in Kenya is a public hospital with 990 beds and two adult medical wards. Patients pay for all medications, diagnostic testing, and care but some have national insurance which covers all of their health expenses. This hospital does not have an infectious diseases or a clinical microbiology specialty service. Clinical pharmacists participate in ward rounds with the clinicians and provide advice on therapy.

The hospital in Tanzania is a referral hospital with 630 beds and four adult medical wards. Patients pay for all medications, diagnostic testing, and care but some have national insurance, which covers all of their health expenses. If the patients cannot afford care, they can receive financial support from the hospital. This hospital has an infectious diseases specialty service that is staffed by one physician with infectious diseases and antimicrobial stewardship training. Pharmacists dispense medications but do not generally provide advice on therapy.

In each of the three study hospitals, physicians were eligible to participate if they were working on the adult medical wards, regardless of medical specialty and level of experience. Physicians were selected for enrollment in the study based on convenience sampling.

### Procedures

The interview guide was developed through collaboration between study team members from Sri Lanka, Kenya, Tanzania and the United States. The interview guide explored current antimicrobial prescribing practices at the participant’s hospital, knowledge regarding antimicrobial resistance and stewardship, and receptiveness to ASP interventions. The first section of the interview guide covered current clinical practices with regards to treating infections; clinical examples were also elicited from the interviewees. The second section covered education regarding antimicrobial resistance and stewardship and current understanding of ASPs. The purpose of this section was to build an understanding of current understanding and beliefs around antimicrobial resistance and ASPs. The third section was dedicated to developing an understanding of local providers’ receptiveness to ASPs.

Interviews were conducted by trained, local research assistants (nurses or doctors) at each site. Interviews were conducted in English, which is the working language by physicians at each hospital, and lasted approximately 45 min. The interviewer followed the interview guide and used open-ended questions with follow-up probes to elicit more detailed information. Each interview was audio-recorded and subsequently transcribed.

### Data analysis

We used applied thematic analysis to analyze the textual data [8]. Two physician researchers initially coded the interviews independently using a set of deductive codes informed by the interview guide. Emergent themes in the interviews were then identified by noting common patterns across the interviews. These were discussed between the two researchers, resulting in a detailed codebook that included both deductive and inductive codes. After completion of independent coding of all interviews by both physician researchers, consensus coding was developed that resolved disagreements between the two physicians. After the initial coding was completed, each deductive code was further categorized—for example, the deductive code of “distrust of diagnostics” was a category created under “use of diagnostics in clinical practice.” The codes were compared between sites based on the number of interviewees at each site who reported similar opinions. NVivo (Version 12) was used to code the interviews.

### Ethical procedures

All interviewed physicians provided written informed consent prior to participation in this study. Ethical approval was obtained from the Duke University Institutional Review Board in the United States, the Ethical Review Committee of the Faculty of Medicine at the University of Ruhuna in Sri Lanka, AMPATH and the Institutional Research and Ethics Committee of Moi University/ Moi Teaching and Referral Hospital in Kenya, the Kilimanjaro Christian Medical College Research Ethics and Review Committee, and the National Institute for Medical Research in Tanzania.

## Results

In total, 45 physicians were interviewed during this study. Table 1 includes details about the study participants.

**Table 1:** Characteristics of interviewed Physicians in Sri Lanka, Kenya, and Tanzania

Characteristics of interviewed physicians			
	Sri Lanka	Kenya	Tanzania
Number of physicians	22	12	11
Age in years, median (IQR)	31.0 (30.0–35.0)	30.0 (29.3–33.3)	34.0 (31.0–38.5)
Time in years since graduation from medical school, median (IQR)	6.0 (5.0–10.0)	5.0 (0.5–8.5)	6.5 (3.5–8.0)

Several themes emerged that were either shared or different across the domains of current practices, barriers to implementation of ASPs, and approach to ASP implementation. Table 2 provides a sample of representative quotations from the interviews for each theme.

**Table 2:** Representative quotes from physician Interviews in Sri Lanka, Kenya, and Tanzania

Current practices	Discrepancies between desired and actual antimicrobial treatment	“I would have loved to actually choose a better or broader [antimicrobial], but they are unavailable, they are expensive for the patients to buy. So you choose what is available and of course it does not eradicate the infection, patients get worse, get septic, go into shock and then they just have mortality.” I-27
		“Sometimes the culture report is sensitive for antimicrobials which are not available in the hospital at the moment. So we have to go with some antibiotics available at that time. And we are usually not asking the patient to buy from outside. So like if the patient is sensitive to meropenem if it is not available, we are using third generation cephalosporin.” I-11
	The role of consultants, pharmacists, and other staff in antimicrobial prescribing	“Luckily for us, as oncology, we have a big team right now unlike in the past one month we have pharmacists who are doing clinical pharmacy and they are very helpful; they are asking very hard questions. Why don’t you give cefepime,…, they are really helping us in making that decision but before that we would just jump on what is available. We try to cover all the aspects be it gram-negative, gram-positive, anaerobes, protozoan we try to put that holistic picture that cover everything.” I-33
		“[Pharmacists] don’t come to the wards. From them we only get to know whether the drugs are available.” -I-22
		“For nurses, because they deal with the patient mostly, at least they have a better, one-on-one with the patient so they tell you this patient has reacted to this drug so please don’t give them.” I-28
		“Nurses are the ones who are monitoring the patients… so once we find that the patient has been having persistent fevers, this is the time when we have to make decisions to broaden our investigations in order to find out what is causing the infection.” I-38
	The impact of the clinical microbiology laboratory and laboratory diagnostics in the prescription of antimicrobials	“The microbiological data are very useful because without any data you will be stuck …you will not be able to treat the patient properly and adequately because you don’t know what you have. The results show you that this is what was found and then it will back-up my prescription and that will help me to be able to give the patient the correct medication.” I-40
		“Although there's been a very recent improvement, part of the challenge again lies in the lab and part of it lies in other places. The fact that we don't have reliable cultures, phlebotomist taught how to do proper blood collection, enough nursing staff to collect samples within a reasonable period of time. There are lots of things that contribute to it, but I think the lab certainly historically has not been a very reliable source of information.” I-34
	Education on antimicrobial resistance and ASPs	“Formally, I have never been trained in programs. If there is any influence it must be from professional discussions in the Department, presentations on the topic. In general I would say there is improvement going on… I-44
		“Ok. Just through case report that’s the most common [form of education] and on my daily rounds you find instances which you find drugs that you expect to be sensitive like ceftriaxone which is most common. Here you prescribe ceftriaxone for a patient with just a UTI, patient does not get better later when you do culture it equally resistant to ceftriaxone which is not expected.” I-24
Barriers to implementation of ASPs	Diagnostic uncertainty and limited awareness of local antimicrobial resistance patterns	“I don't think we get as much data back. So it's mostly anecdotal, certainly there've been resistant gram negatives. They are certainly concerned about MRSA (methicillin resistant *Staphylococcus aureus*), but I've never actually seen a positive culture for it. It's really unfortunately hard to even approach this from an institutional perspective given the lack of data.” I-34
		“I don’t know that [if the resistance patterns vary with wards]. Since there has been no research done about it, no research no right to speak. So I am not so sure.” I-38
	Local clinical practice and customs regarding antimicrobial prescribing	“You know what is practiced for so long is hard to break, so even when you come in you say okay, so maybe a lot of people have been using a certain antibiotic throughout, you know, and it is a first line, so you have to look at different factors. So that is a big barrier. Or you find that in another department they have already started on antibiotics that are not appropriate. So those are cultural traditional use of antibiotics that has to be broken in that system so there is a need for educating the people” I-43
	Unrestricted access to antimicrobials	“Very strong drugs are given over the counter that’s why nowadays we have very simple diseases that are resistant to the first line [antimicrobials], so we are left in such a difficult position in our prescription so I think that is where we need to follow and address, that is one of the challenges.” I-25
		“Antimicrobials are available in the pharmacy in town without a prescription. So that's a major challenge I think for antimicrobial resistance, in casualty (on call) when patients present, I think they're often getting antimicrobials before a diagnostic workup, including cultures have been taken, and therefore makes it much harder to figure out what we're treating in the long run. So we're stuck with the empiric guesses and sometimes that leads to inappropriate continuation of antibiotics or broad spectrum of antibiotic use. That's broader than it needs to be.” I-34
Approach to ASP implementation	Creation of guidelines	“It will help us come up with a guideline for what antibiotics to use at what point instead of just people dispensing antibiotics freely without really waiting for lab results to come back. In that way we will reduce occurrence of antimicrobial resistance. “ I-28
	Improving clinical documentation	“If I’m not available if somebody has to see the patient in clinical emergency they should be aware of why this person has started this [medicine]. So it’s really important to document.” I- 22
		“The benefits will be many. Because you know this unnecessary prescribing will make doctors more aware that if I do this, someone will ask me… Why did I do this? So even the thought process will increase that instead of just giving an antibiotic, you’re thinking… Where; why this antibiotics will cover this area… And it will also help us know which antibiotics are right, you know, in collaboration with the stewardship program.” I-43
	Structure of ASP leadership	“I absolutely agree that pharmacists have a major role to play in antibiotic stewardship again because they, they can serve as some of the link between the lab and the available formulary. And help advise the formulary actually and to also help limit a broad-spectrum antibiotic use, one that's not necessary.” I-34
		“No they can’t, pharmacists will not decide which antibiotic to use. But they will provide information regarding which antibiotic available here and the drug doses and other things.” I-33
		“Because a microbiologist is someone who has specialized in hospital microbiology so the expertise they have will go a long way in improving patient outcomes because of influencing my judgment strongly positively and in deciding what drugs to use so their input is very important.” I-30
	Improving education on antimicrobial resistance and stewardship	“I think all prescribers at whatever level should undergo antibiotic stewardship training to enhance their knowledge on antimicrobials and to make sure that there is rational use of antibiotics, I think it's an important program and it should happen periodically, I think the other challenge is that it happens once in a while and you know that you are here for a time, once you go that is it, so it should happen periodically maybe quarterly or at some point.” I-32

### Current practices

#### Discrepancies between desired and actual antimicrobial treatment

Respondents noted a discrepancy between the treatment they desired to prescribe and the one they actually administered. At all three sites, high cost and limited availability were the major drivers of discrepancy between desired and actual antimicrobial treatment. Some physicians described situations in which they were stuck between the decision of treating with an antimicrobial that may not work given an organism’s susceptibility pattern versus not treating at all due to lack of access to antimicrobials to which the organism appeared susceptible.

“Sometimes the culture report is sensitive for antimicrobials which are not available in the hospital at the moment. So we have to go with some antibiotics available at that time. And we are usually not asking the patient to buy from outside. So like if the patient is sensitive to meropenem and if it is not available, we are using third-generation cephalosporin.” I-11

Similar statements regarding cost as a barrier to prescribing desired antimicrobial agents was shared by 4/22 physicians in Sri Lanka, 4/12 in Kenya, and 5/11 in Tanzania. In interviews at all study sites, prohibitive cost of antimicrobials both during admission and after discharge were discussed. Even in the hospital in the public hospital in Sri Lanka, when antimicrobials were not available in the hospital, patients were asked to purchase them from the private sector.

“Most of the time they are not available in the hospital setting, freely available. Sometimes the patient cannot afford for a private sector. We ask the patient but they cannot afford. In that case we are going to shift to another drug which is similar or same class of antibiotic” I-10

Limited availability was mentioned by 9/22 physicians in Sri Lanka, 9/12 in Kenya, and 4/11 in Tanzania. Prior exposure to antimicrobials from either local pharmacies or clinics had an impact on the choice of antimicrobials used after patients were admitted, per one interview each in Kenya and Tanzania. Poor drug quality of meropenem was listed as a reason for discrepancy between desired and actual treatment in 2/22 of the interviews in Sri Lanka but was not mentioned in either Kenya or Tanzania.

#### The role of consultants, pharmacists, and other staff in antimicrobial prescribing

Overall, many physicians sought specialist advice, when available, from the microbiology or infectious diseases services for the management of complicated cases. The recommendations from specialists regarding antimicrobial prescription was followed by the clinicians who requested the advice at all study sites. Only 2/22 participants in Sri Lanka expressed concern over specialist input as they felt the consultants did not have as much clinical context as they did. “Sometimes they are not clinically relevant in my personal opinion.” I-3. This concern was not raised in Kenya or Tanzania.

Opinions regarding the role of clinical pharmacists in antimicrobial prescribing varied among the three sites. In the hospital in Kenya, clinical pharmacists rounded with the ward teams. As the pharmacists were in frequent contact with physicians, pharmacists’ opinions on prescribing seemed to be more respected than in other locations. “A very big role. I must congratulate the hospital has set up clinical pharmacy here so that at least every team, most teams have a pharmacist and it plays a big role and it really assists in patient management.” – I-23.

In the hospital in Sri Lanka, where there is no contact between pharmacists and physicians during daily clinical work, the value of pharmacists’ input was not as apparent to the physicians. “[Pharmacists] don’t come to the wards. From them we only get to know whether the drugs are available.” -I-22.

The importance of nursing staff in antimicrobial prescribing varied among the sites as well, but physicians from all three sites recognized the important role that nurses have in monitoring patients’ clinical status and in the delivery of antimicrobials. The value of nurses in monitoring patients and delivering antimicrobials was mentioned in 4/22 interviews in Sri Lanka, 9/12 in Kenya, and 5/11 in Tanzania.

#### The impact of the clinical microbiology laboratory and laboratory diagnostics in the prescription of antimicrobials

Results from microbiological cultures were felt to be very helpful for tailoring antibiotic courses. However, some physicians were concerned with the reliability of the culture results from their microbiology laboratories. There was concern that cultures may be contaminated when they were obtained, resulting in mixed bacterial growth that was difficult to interpret. Also, others mentioned distrust in the microbiology laboratory when cultures were negative, even when infections were highly suspected. Some remarked that most clinical decisions were made before culture results were available. These concerns may impact the ability of providers to narrow the spectrum of antimicrobial treatment.

“So maybe I don’t know if there are other modalities of doing culture or maybe the way people are taking the cultures may be the sterility is not perfect or the growing of the organisms… Because sometimes you might find that your patient has fever or other signs of infection, but the culture comes out negative… You take another culture but it comes out negative also. Sometimes you are not really sure. There is human error or lab error which can happen.” -I-36.

In addition to discussing bacterial cultures, the use of biomarkers in clinical decision-making was considered. Erythrocyte sedimentation rate (ESR) or C-reactive protein (CRP), two biomarkers which are sometimes used to identify the presence of infection, were generally thought to not provide much additional evidence for physicians to start antimicrobials as they were markers of inflammation and too non-specific to have an impact on antimicrobial use. “Chronic diseases, inflammatory diseases, auto-immune diseases, almost anything can raise the ESR, so it’s not anything that I would use to start antibiotics based on ESR” -I-43.

Procalcitonin, a newer biomarker that is sometimes used to differentiate between bacterial and viral infection, was not available at any of the three sites. While most physicians felt procalcitonin would be helpful if available, 9/22 participants in SL, 5/12 in Kenya, and 5/11 in Tanzania were uncertain if procalcitonin would be helpful mostly given limited (or no) experience with its use clinically. Four participants felt procalcitonin would not be helpful in determining if antibiotics were needed (2 Sri Lanka, 1 Kenya, 1 Tanzania). Reasons cited included that procalcitonin is not specific for bacterial infection, is a prognostic not diagnostic marker, and is too expensive.

#### Education on antimicrobial resistance and ASPs

Most participants at all three sites stated that their training in antimicrobial resistance occurred in medical school. “Obviously antimicrobial resistance is something you learn in school… It is just book knowledge what you were taught in medical school. Now on practice point when you are seeing patients you suspect antimicrobial resistance because, maybe your patient is not responding to the therapy you are giving.” - I-29.

Many physicians stressed the important role of clinical training or work experience. “Clinical experience is mostly important, for example I have seen most of the junior doctors, and they used antibiotics unnecessarily. But with their experience you can learn most of the infections are due to viral infection which does not need antibiotics.” -I-13.

One participant mentioned reading articles online and on Up-To-Date to learn more about antimicrobial prescribing practices. “Well, mostly is my own readings from Up-To-Date, and do I had any, maybe once, not a recent though, CME; mostly from my own readings, from Up-To-Date and CMEs.” -I-32.

In total, 19/22 respondents in Sri Lanka, 8/12 in Kenya, and 7/11 in Tanzania had not heard of ASPs before these interviews.

### Barriers to implementation of ASPs

#### Diagnostic uncertainty and limited awareness of local antimicrobial resistance patterns

Most participants at all three sites were not aware of the antimicrobial resistance patterns at their hospitals or of resources available to find this information.

“I don't think we get as much data back. So it's mostly anecdotal, certainly there've been resistant gram negatives. They are certainly concerned about MRSA, but I've never actually seen a positive culture for it. It's really unfortunately hard to even approach this from an institutional perspective given the lack of data.”-I-34

However, one respondent in Sri Lanka was able to discuss different antimicrobial-resistant infections that were felt to be more prevalent in certain wards. “In orthopedic wards, MRSA is a common problem. In the ICU, resistant *Acinetobacter* ventilator-associated pneumonia is difficult to control with antibiotics because of the resistance. In the medical ward ESBL UTIs… Those are the resistant bacteria I’ve heard of in our hospital.”- I-17.

#### Local clinical practice customs regarding antimicrobial prescribing

Local traditions regarding antimicrobial prescribing practices and workflow were cited as a potential barrier to the future implementation of ASPs by 12/22 in Sri Lanka, 4/12 in Kenya, and 3/11 in Tanzania. Some cited concerns about the extra time that documentation would take given the busy clinical services. Others felt that many would resist the changes or recommendations from ASPs as these would often require physicians to change their normal practices. “But I think the biggest thing is to break the traditions and to teach the people on which antibiotics are best and not just to prescribe one type of antibiotic to everyone you see.” – I-43.

#### Unrestricted access to antimicrobials

As discussed in ‘Current Practices’ above, drug availability was mentioned as a potential barrier to ASPs by participants in all three countries. Over-the-counter use or use in private facilities was also felt to be an impediment to rational use of antimicrobials.

“Mainly in the private sector... Because in Sri Lanka we don’t have the general practitioners who are established. So, a lot of the MBBS doctors will be doing the private practice at different places and there what the people want is some quick response to the symptoms they have. So generally, the practice there is of giving high-power antibiotics at the initial stage of disease. That of course conflicts very badly with our antimicrobial practices through Sri Lanka.” - I-21

### Approach to ASP implementation

#### Creation of guisdelines

The creation of guidelines was a recommendation raised by interviewees, without prompting, at all three sites (13/22 in Sri Lanka, 7/12 in Kenya, 5/12 in Tanzania) as a strategy to improve the use of antimicrobials in the hospital setting. “I think we should prepare our own guidelines for our hospital with the help of the microbiologist and whole medical team. All the consultant physicians, MOs (medical officers) in pediatrics and medicine and we should make our own guidelines and these guidelines should be evaluated at least in 3 months, 6 months at least annually and change our prescription pattern.”-I-13.

#### Improving clinical documentation

Documentation of the reasons for prescribing antibiotics can improve communication between physicians and other staff. Documenting the justification will also give physicians another opportunity to think through their reasoning for using a given antimicrobial. Most participants at all three sites were receptive to documenting reasons for prescribing antibiotics in the chart. “The benefits will be many. Because you know this unnecessary prescribing will make doctors more aware that if I do this, someone will ask me… Why did I do this? So even the thought process will increase that instead of just giving an antibiotic, you know, you’re thinking… Where, why these antibiotics will cover this area… And it will also help us know which antibiotics are right, you know, in collaboration with the stewardship program. So the advantages are many.”—I-43.

However, some noted the high clinical volume as a potential impediment to thorough documentation.

“It is important but impractical, given the conditions we work here, we usually encounter around 50 to 70 patients in a casualty (on call) night. So, it is not practical to document everything though it’s ideal.”- I-14

#### Structure of ASP leadership

Views on who should be involved in ASP leadership mirrored views on current practices regarding advice from consultants, pharmacists, and other staff. In all three countries, participants valued recommendations from specialists and stated they would take advice from physicians involved in ASP leadership. However, there were mixed views on the opinions of clinical or hospital-based pharmacists. All of the participants in Kenya were receptive to pharmacist input (12/12), all but one in Tanzania were receptive 10/11, but 12/22 in Sri Lanka opposed.

#### Improving education on antimicrobial resistance and stewardship

Improving education regarding antimicrobial prescribing and resistance patterns was a non-elicited strategy mentioned in all three countries in the development of ASPs (7/22 in Sri Lanka, 6/12 in Kenya, 6/12 in Tanzania). Many interviewees did not limit the education only to physicians, but also included pharmacists and nurses. “It is mainly about knowledge and information that should be provided to all the medical personnel, so even the nurses, doctors, the pharmacists… these should be also involved in the prescribing of antibiotics and not just writing unnecessarily.”-I-37.

## Discussion

We conducted a multi-site qualitiative study in tertiary medical centers in three LMIC to determine the barriers to and feasibility of ASPs in a context-sensitive manner to inform our implementation of ASPs in these centers. The interviews shared many similarities between sites. A common theme was the lack of prior knowledge of ASPs accompanied by the willingness to participate if programs were developed locally. Additionally, the interviews highlighted a lack of adequate diagnostic capabilities, availability of specific antimicrobials and frustration over costs of medications.

Our study identified the need for improved access to a variety of antimicrobials, as physicians were frequently forced to treat infections with antimicrobials on hand, even when the organisms causing infections were resistant to the antimicrobials. Lack of access to antimicrobials is an issue shared in other LMICs as well. One study of Nigerian physicians suggested similar concerns in lack of access to antimicrobials when developing ASPs locally [9]. Ensuring quality antimicrobials is important as well. A meta-analysis peformed in 2018 indicated a prevalence of 13.6% in substandard or falsified antimicrobials in LMICs [10]. The low quality of meropenem available through the public healthcare system was discussed by multiple physicians in Sri Lanka. Counterfeit or substandard medications that have lower levels of active drug than labeled pose a risk for future antimicrobial resistance [11]. In a 2020 review of the 2013 Lancet Commission on antimicrobial resistance, little progress in the surveillance for counterfeit or sub-standard antibiotics had occurred in the interim [12].

Empiric antimicrobial use was frequently attributed to long turnaround times from the microbiology lab. Lack of diagnostic capabilities and distrust in microbiological culture results was a common theme raised by physicians. When discussed with members of our team to clarify reasons for distrust in microbiology results, team members stated that many patients at their centers were transferred from other hospitals, having already had long hospitalizations, antibiotics, and limited to no microbiologic testing. In these situations, providers often feel there is little utility to collecting blood cultures and empirically place patients on broad-spectrum antibiotics. In other studies, physicians have attributed the overuse of antimicrobials to lack of diagnostic capability [13]. In a qualitative study performed in a secondary level hospital in India where physicians and clinical pharmacists were interviewed, unreliable antibiograms were described as a challenge in implementing stewardship programs. As many of the physicians used empiric antibiotics, and cultures were only drawn when patients were not responding to therapy, the sensitivities from cultures were likely skewed toward higher resistance than was truly present within the hospital [14]. Improved laboratory capacity and structured surveillance techniques to create an antibiogram is a critical need in many LMICs to improve antimicrobial prescribing. In 2018, the WHO published a list of essential diagnostic tests, which included microbiologic cultures and antimicrobial susceptibilitiy testing [15]. However, improvement in diagnostic capabilities needs to be accompanied by “building trust” between the diagnostic laboratories and physicians. In a study of physicians’ perceptions regarding the utility of laboratory diagnostics in Ghana, despite high laboratory capacity, local providers remained reliant on clinical judgement and empiric therapy [16]. Improving the trust of clinical providers in the results provided by the microbiology laboratory could help improve prescribing behaviors. Representation of clinical microbiology varies greatly in ASP leadership. However, their inclusion would enhance the communication between the laboratory and clinical care [17]. Improving communication could help increase trust in microbiology among clinicians. Summary data on commonly identified bacteria and their antimicrobial resistance patterns within each hospital could be used to guide the choice of empiric antimicrobials. In addition, local susceptibility and culture data could inform the development of antimicrobial guidelines. The development of a local antibiogram and local guidelines could be key actions targeted by ASPs at these LMIC hospitals.

Widespread (or easily accessible) hospital-level antimicrobial treatment guidelines are not available for physicians in either Tanzania or Kenya. The providers in these countries occasionally would look to other resources or guidelines to determine the appropriate antimicrobial therapy. As resistance rates differ between countries, this could lead to inappropriate use of antimicrobials in different clinical situations. The Infectious Diseases Society of America (IDSA) recommends the creation of guidelines based on local resistance patterns to improve the utilization of antimicrobials [18].

Education would also be essential in the introduction of ASPs in these hospitals. The majority of participants at all three sites had never heard of ASPs previously and routine practices recommended by ASPs did not seem to be a part of their daily prescribing habits. Most participants cited medical school as the last time they had received formal training regarding antimicrobial resistance and antimicrobial prescribing practices. Strategies for improving education regarding antimicrobial resistance and stewardship practices range widely from short didactic sessions to bedside stewardship rounds to dedicated continuing medical education sessions on antimicrobial resistance and stewardship [19]. Education for pharmacists would also be an important component of ASPs. A study assessing the knowledge of Australian pharmacy students compared to Sri Lankan pharmacy students showed a lower level of knowledge about antimicrobial resistance among the students from Sri Lanka [20].

Another important factor for the implementation of ASPs in these hospitals woud be determining the leadership structure for such programs. In the site in Kenya, a team comprised of both pharmacists and physicians would likely be effective based on opinions expressed in the Kenyan interviews, as pharmacists are frequently involved in clinical care. However, in both Tanzania and Sri Lanka, physicians did not feel the pharmacists should play a large role in antimicrobial prescribing decisions. These opinions seemed to correlate with the amount of contact the physicians had with pharmacists on the unit floors or in daily practice. Based on our interviews, we believe that leadership of ASPs in the sites in Tanzania and Sri Lanka would both benefit from strong physician leadership. However, further education of physicians regarding the diverse roles of other staff in antimicrobial prescribing, as well as encouraging pharmacists to engage more closely in clinical care, may also be beneficial. Ultimately, we believe that ASPs should be multidisciplinary teams.

There were several limitations in this study. First, this study was limited to a small number of participants at three hospitals in three LMICs. Extrapolation to national trends even within these countries would not be possible nor would extrapolation to all LMICs. The participants of the interviews were all physicians in medicine or medicine subspecialties. Opinions of surgeons, pharmacists, or other hospital staff were not elicited and may have differed significantly from the opinions expressed in these interviews. These interviews were all performed in tertiary care centers, and themes relevant to other levels of care are likely not highlighted in these interviews. For example, many physicians in our study discussed the widespread availability of antibiotics in community pharmacies. However, perspectives from community pharmacy and primary care providers are notably missing in this analysis; such perspectives may have raised themes such as poor access to licensed physicians being a driver of antibiotic use in the community [21].

## Conclusion

In conclusion, views shared between the three sites on current barriers to appropriate antimicrobial prescribing included lack of availability or prohibitive cost of antimicrobials, distrust in results from the microbiology lab, and resistance to change from established, or traditional, practices. Next steps for the implementation of ASPs in these hospitals should include widespread educational programs for physicians and staff, the creation of hospital-specific guidelines for appropriate antimicrobial use, and the development of multidisciplinary ASP teams that eventually include representatives for physicians, pharmacists, and microbiologists.

## Data Availability

The datasets used and/or analyzed during the current study are available from the corresponding author on reasonable request.

## Abbreviations

ASPs: Antimicrobial Stewardship Programs
CDC: Centers for Disease Control and Prevention
LMICs: Low- and middle- income countries
MBBS: Bachelor of Medicine, Bachelor of Surgery
MO: Medical Officer
WHO: World Health Organization
